# Dr. Kinglake, in Reply to Dr. Kentish, on the Treatment of Burns or Scalds

**Published:** 1806-09

**Authors:** Robert Kinglake

**Affiliations:** Taunton


### Dr. Kinglake, in Reply to Dr. Kentish, on the Treatment of Burns or Scalds.

*To the Editors of the Medical and Physical Journal.*

Gentlemen,

THE question lately agitated between Mr. Harrold, Dr. Kentish, and myself, relative to the comparative advantage of the cooling and heating mode of treating burns and scalds, is not likely to be soon set at rest by the positive manner in which Dr. Kentish would enforce his opinion. Your last Journal contains some comments of Dr. K. on the effects of the cooling treatment. In those, he feels no hesitation in admitting the apparent but delusive benefit of refrigerating applications, confidently ascribing the seeming advantage to the healing powers of nature. "Of this fact," says the Doctor, (alluding to the supposed virtue of cold water) "I shall not for a moment doubt; but what does it prove? Merely, that burns or scalds, at a certain extent, will get well in defiance of the application of cold water. It is not in these accidents alone I have known nature triumphant over every officious interference of art, which have retarded her progress in re-establishing the equilibrium of health." No fact, no truth, can pretend to cope with such a licence as Dr. K. has here assumed. However speciously appearances may indicate usefulness in a mode of remedy, yet no real benefit has been rendered!

ed! and why? because it would be repugnant to a given principle that theoretically denies the possibility of such an occurrence! This is surely arrogating too much; it divests much too sweepingly cold water, of the power of relieving inflammatory heat by its refrigerating influence, to transfer the capability of benefitting to a "principle," that exclusively prescribes additional excitement as the only rational mode of rendering service.

Can Dr. K. justly complain of having either his Essays or the practice they would inculcate, neglected, when he proposes the exclusive power of his remedy, in such unqualified terms of heavy censure, on that which both medical and popular experience has abundantly sanctioned?

How will Dr. K. feel at being told, that the very cases on which he rests the demonstrative proof of the superior, nay, the infallible efficacy of his remedy, have really amended under the restoring aid of nature, independantly of the "officious," the painful "interference of his art." Such latitudinarianism in resisting the evidence of facts, would furnish an equal objection to every medicinal effect that could be produced. If instead of untying the knot that may obstruct the solution of a difficulty in medical inquiry by rational explanation, it be thus abruptly cut, it may be justly feared, that an end would soon be put to all patient and correct investigation.

The inflammatory violence inflicted by fire will no doubt, in many instances, spontaneously recover; but if this be ultimately accomplished through the wearisome and painful processes of protracted inflammation, ulceration, and cicatrization, is no benefit rendered by an application that obviates these embarrassments and expedites the desired cure? This, cold water accomplishes in the most evident, commodious, and satisfactory manner. It will then be to no purpose for Dr. K. to expect that oil of turpentine is the true and only remedy for a burn or scald, that it owes its virtue to its stimulating influence, and that therefore, *è converso*, cold water by its sedative effect must be injurious.

Popular remedies are not often popular evils; the multitude may be thoughtless, but they are attentive to matters of fact, and will not be cajoled into a persuasion, that either the removal of pain is its aggravation, or that freedom from all danger of losing life is actually verging on death. The untutored can feel and express sensation with sufficient correctness, to dispute with the medical theorist on the subject of pain. It requires no erudition to judge

P 2 of of the effect of a remedy, when the first step of its influence is to procure an alleviation of torture. This is the experience which mankind very generally can furnish in support of the efficacy of cold water in burns or scalds.

The case of Ann M'Quicker, recited by Dr. K. is of the description that often proves mortal in defiance of any aid that can be afforded. The extent and severity of the burn must have so tortured and convulsed the system as to have kept life as it were on the wing of departure. But what had induced all this exhaustion of vital power, this chilling tremor? Surely, the agonising pain consequent on the burn. Would it not then have been advisable to have reduced the inflammatory action, and to have alleviated the threatening pain by cooling applications, rather than to have exasperated it by those which were heating? Why was laudanum given? Was it not with a view to diminish the concomitant irritation? But why question a fact? Dr. K. may fairly demand, can you refute it? No, it is not my wish so to do; but it would be easy to avail myself of Dr. K's casuistry, in objection to its validity, by asserting that it was one of those instances in which "nature is triumphant over the interference of art." The burn was, it must be confessed, deplorably severe; but on what ground can the eventual cure be held up as vastly transcending what could have been expected from any other mode of treatment? The accident occurred on the 11th of January, and the patient was not "discharged cured until the 11th of May!" A period sufficiently long to exclude the boasted advantages of the treatment from any extraordinary merit for rapidity of success, and quite long enough for "nature to have been triumphant over the interference of the art," whether "officious" or not, which was adopted.

Dr. K. remarks, that "had Dr. Coxe entertained the same ideas with Dr. Kinglake, I have little doubt but all vital action would have been extinguished in a short time, and it would then have ranked with the many cases we hear of, where the patient is dreadfully burnt, and dies in a few hours." Dr. K. is at liberty to entertain but little doubt respecting what would have been the event, if my ideas of appropriate treatment had been practically adopted; but his so doing, does not clear the question of all ambiguity. Less excitement would have perhaps advantageously economised the powers of life, and not have wasted them in the profuse manner that must have happened from the stimulating means which were employed.

Under

Under these circumstances, both less immediate danger and distant distress from slow and difficult healing might have been the happy consequence. But is this case deemed the *experimentum crucis* of the infallibility of the stimulating treatment? Could nothing worse, compatible with life, possibly occur for a trial of its curative powers? Is this the description of case which Dr. K. would pronounce to be necessarily mortal if treated by any other mode than that which he proposes? If so, he would do well to retract that declaration, as it is highly probable that the use of topical cold would rescue such cases from all danger, and that much more speedily, and less painfully, than could be effected by the heating method of treatment.

Dr. K. then says, that "the danger of suddenly checking an increased action may be observed in a variety of circumstances; the taking a draught of cold water when the body is heated, occasions sometimes the most dreadful effects, such as dimness of sight, staggering, difficult respiration, coldness of the extremities, imperceptible pulse, and, in the course of a few minutes, death." These effects are not correctly referable to diminishing increased action, but to the chilling influence which the swallowing of a cold fluid would have when in a state of perspiration. It would be nature's most salubrious cordial in circumstances of increased action *only,* unattended with the cooling process of profuse sweating. The dry and heated skin from febrile commotion, or from any other cause, inducing violent arterial action, is most beneficially removed by a draught of cold water; but a moist skin makes all the difference between the same agent proving a direct remedy and a deadly poison.

Dr. K's principle and practice shall receive from me all the investigation their importance deserves; and if they appear to be as well founded as Dr. K. conceives they are, I shall feel no difficulty in acceding to their high merit, and should esteem it unjust to attribute such glaring proofs of virtue as Dr. K. has thought proper to do with respect to the use of topical cold, to the exclusive efficiency of nature. Dr. K. may rely on the purity of my intention, and likewise on my disposition to prefer the greater good, if even it should be found to consist in the practice which disclaims the cooling treatment as "an officious interference of art;" though, in my present opinion, it is in all cases of burns or scalds, under certain restrictions, the most appropriate and effectual remedy that can be devised for such injuries. I am, &c.

*Taunton, July* 19, 1806. ROBERT KINGLAKE.

